# Gamma-Irradiated Non-Capsule Group B Streptococcus Promotes T-Cell Dependent Immunity and Provides a Cross-Protective Reaction

**DOI:** 10.3390/ph16020321

**Published:** 2023-02-20

**Authors:** Yong Zhi, Fengjia Chen, Guangxu Cao, Fang Li

**Affiliations:** 1 Department of Obstetrics and Gynecology, Shanghai East Hospital, School of Medicine, Tongji University, Shanghai 200092, China; 2 Research Division for Radiation Science, Korea Atomic Energy Research Institute, Jeongeup 56212, Republic of Korea

**Keywords:** Group B Streptococcus, inactivated vaccine, Th1/Th17, cross-protection, BMDC

## Abstract

Group B Streptococcus (GBS) is a Gram-positive bacterium commonly found in the genitourinary tract and is also a leading cause of neonatal sepsis and pneumonia. Despite the current antibiotic prophylaxis (IAP), the disease burdens of late-onset disease in newborns and non-pregnant adult infections are increasing. Recently, inactivation of the pathogens via gamma radiation has been proven to eliminate their replication ability but cause less damage to the antigenicity of the key epitopes. In this study, the non-capsule GBS strain was inactivated via radiation (Rad-GBS) or formalin (Che-GBS), and we further determined its immunogenicity and protective efficacy as vaccines. Notably, Rad-GBS was more immunogenic and gave rise to higher expression of costimulatory molecules in BMDCs in comparison with Che-GBS. Flow cytometric analysis revealed that Rad-GBS induced a stronger CD4^+^ IFN-γ^+^ and CD4^+^IL-17A^+^ population in mice. The protective efficacy was measured through challenge with the highly virulent strain CNCTC 10/84, and the adoptive transfer results further showed that the protective role is reversed by functionally neutralizing antibodies and T cells. Finally, cross-protection against challenges with prevalent serotypes of GBS was induced by Rad-GBS. The higher opsonophagocytic killing activity of sera against multiple serotypes was determined in sera from mice immunized with Rad-GBS. Overall, our results showed that the inactivated whole-cell encapsulated GBS could be an alternative strategy for universal vaccine development against invasive GBS infections.

## 1. Introduction

Group B Streptococcus (GBS) is an encapsulated Gram-positive opportunistic pathogen that colonizes the gastrointestinal and/or genitourinary tracts of approximately 25% of healthy individuals. Epidemiological and clinical studies have found that GBS is the leading cause of life-threatening bacterial infections in newborns and infants [1,2]. In neonates, invasive GBS diseases are classified as early-onset (0–7 days of life) and late onset disease (7–90 days of life), and can result in sepsis, pneumonia, meningitis, and even death [3,4]. Every year, there are >410,000 estimated cases of GBS maternal and fetal infections worldwide, resulting in 147,000 stillbirths and infant deaths [5]. Although intrapartum prophylaxis (IAP) can prevent early onset disease, it does not alleviate the burden of late-onset disease and maternal GBS infections [6,7]. Moreover, the incidence of invasive GBS infections in non-pregnant adults, particularly among immunocompromised individuals and the elderly, has continued to grow [8]. The application of effective GBS vaccines is the most promising method for preventing GBS.

The development of GBS vaccines for maternal use has been identified as a priority for the World Health Organization (WHO) as a preferred product [9]. Due to the presence of capsular polysaccharides, similar to those of other encapsulated bacteria, such as *Streptococcus pneumoniae* and *Hemophilus influenzae*, GBS can evade the host immune system, making it an important vaccine target owing to its serotype-dependent antigenicity [10,11]. GBS can be divided into ten types (Ia, Ib–IX) based on the structurally different capsular polysaccharides and their independent immunogenicity [12]. A polysaccharide-conjugated vaccine (PCV) developed by GlaxoSmithKline has been investigated in a phase II clinical trial as a trivalent GBS vaccine against serotypes Ia, Ib, and III (NCT02046148), and serotype-specific antibodies were detected via a multiplex immunoassay at 30/60 days post-vaccination [13]. Recently, a novel hexavalent conjugated vaccine (GBS6) developed by Pfizer has been investigated in several clinical studies [14]. Clinical studies showed that these PCVs only induced serotype-specific serum IgG antibodies capable of protecting against relevant GBS serogroups. However, serotype switching and the geographic serotype distribution of prevalent GBS strains should be considered. Notably, approximately 8–14% of clinical isolates have been classified as non-typeable strains [15]. Taken together, these results will be of concern for the implementation of polysaccharide-based vaccines [16].

The complex pathogenesis of GBS infections arises from bacterial virulence factors, which vary among strains and are differentially expressed in response to the host and underlying conditions [7]. A universal GBS vaccine candidate should give rise to the production of functionally active antibodies and cell-mediated responses against all GBS serotypes. Nevertheless, inactivated whole-cell vaccines have demonstrated favorable safety profiles and are used widely against many bacterial pathogens. These vaccines can induce protective immune responses because they contain multiple surface-conserved antigens [17,18]. Formalin-killed pathogens, such as *Vibrio cholerae*, poliovirus, and Japanese encephalitis virus, have been used in licensed inactivated vaccines. However, the reduced immunogenicity due to formalin-induced cross-linking contributes to irreversible modifications of antigenic epitopes, resulting in short memory immunity and a lower level of protection [19]. Additionally, formalin-inactivated vaccines can cause side effects, including tissue eosinophilia and allergic contact dermatitis [20]. Thus, the identification of a safe and effective method of inactivation will be valuable for research on inactivated vaccines. 

Ionizing radiation is the transmission of energy in the form of a particle wave, and can be used for the sterilization of medical devices, in the food industry, and in plant breeding [21,22,23]. Compared with heat and chemicals, radiation can induce DNA breaks in viruses and bacteria through energy deposition, while maintaining the integrity of antigenic proteins [24]. Furthermore, irradiation is easy and cost-effective to produce without generating toxic hazards or chemical residues, excluding the induction of allergic responses [18]. In the early 1970s, gamma radiation was used to inactivate the Venezuelan equine encephalitis virus and develop an inactivated vaccine [25]. A gamma-irradiated influenza A vaccine has been proven to be more effective at activating effector CD8^+^ T cells and providing long-lasting protection against heterologous influenza viruses [26,27]. A previous study suggested that gamma-irradiated *Brucella* bacterial vaccines could prevent replication, retain metabolic activity, and generate a strong immune response against extracellular and intracellular pathogens [28]. Recently, gamma-irradiated *S. pneumoniae* was shown to markedly increase Th17 CD4^+^ T cell activation via dendritic cells, suggesting its potential role as an effective pneumococcal vaccine candidate [29,30]. However, the latent capacity of irradiated GBS to enhance humoral or cellular immune responses remains unclear.

In the present study, we evaluated the immunogenicity and protective efficacy of gamma-irradiated non-capsulated GBS (Rad-GBS) compared with those of formalin-inactivated GBS (Che-GBS) as a universal GBS vaccine candidate. This study investigated whether Rad-GBS elicited a higher humoral response and induced proliferation of CD4^+^ IFN-γ^+^ and CD4^+^IL-17^+^ T cells compared to Che-GBS. Additionally, vaccination with Rad-GBS should provide cross-protection against multiple serotypes of GBS strains in a mouse meningitis model. We demonstrated the rapid and complete eradication of the GBS genome via simple gamma radiation, while maintaining highly diverse surface epitopes for the induction of T cell-dependent immune responses. This approach may offer a method for the development of a novel, universal, and inactivated GBS vaccine. The Group B streptococcus strains used in this study was showed in Table 1.

## 2. Results

### 2.1. Surface Protein Epitope Integrity in Rad-GBS and Che-GBS

**Figure 1:**
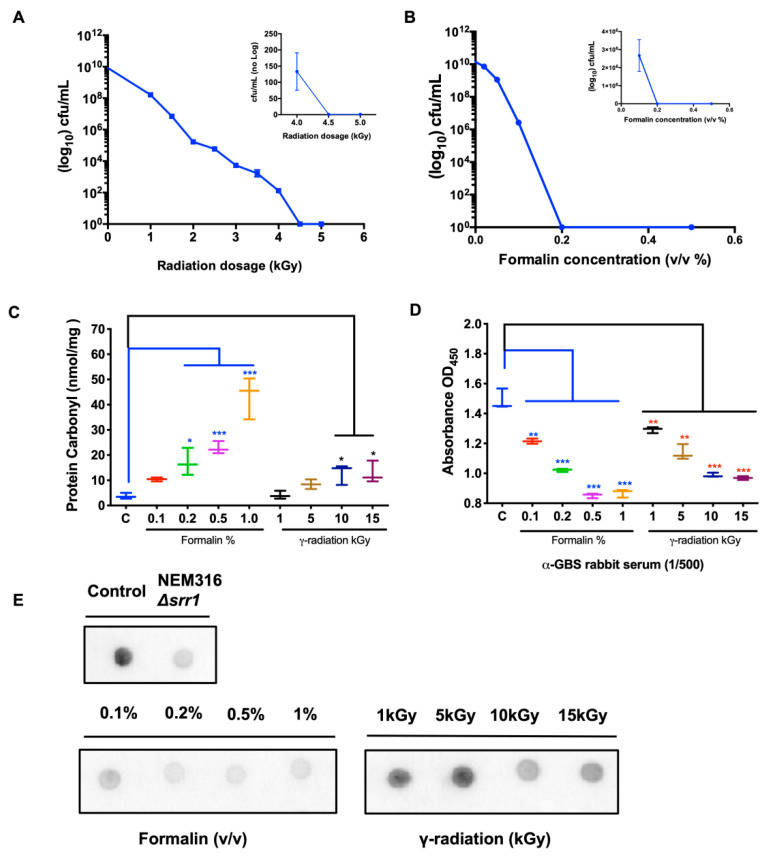
Reduction of the viability of Group B streptococcus treated with gamma-radiation or formalin. Clinical non-capsule GBS isolate NSP14-358 was exposed to various doses of (**A**) gamma radiation or concentrations of (**B**) formalin in PBS for 2 h at 37 °C. Bacterial viability was determined by counting CFUs on blood agar plates. The aliquots of bacteria grown in the exponential phase were treated with various concentrations of formaldehyde or doses of gamma radiation; the non-treated was GBS used as control. (**C**) The levels of protein carbonyl (nmol/mg) in inactivated GBS lysates were used to quantify the damage to protein epitopes according to the commercial kit. (**D**) The antibody-binding affinities of inactivated GBS were assessed through ELISA using diluted rabbit anti-GBS serum (1/500 dilution). (**E**) The binding with surface SRRN2N3 epitopes of the antibody by inactivated GBS samples was tested via a dot plot. All data are expressed as the mean ± standard deviation of triplicate samples or three independent experiments, and p-values were determined by performing an unpaired t test. *** Stands for *p* < 0.001, ** stands for *p* < 0.01, and * stands for *p* < 0.05 and NS for not significant.

We reported clinical isolate GBS NSP14-358, excluding capsular polysaccharides, as a vaccine strain for inactivation [36]. The approach of generating inactivated whole-cell GBS vaccines through gamma irradiation or formaldehyde was described previously [37]. Generally, excessive modification via over-inactivation, regardless of the inactivation method, damages the antigenicity of epitopes, thereby reducing vaccine immunogenicity [38]. To identify the optimal inactivation conditions, the bacteria were harvested and treated with various doses of gamma radiation or formalin. Dosage-dependent survival curves suggested that all bacteria were eliminated sufficiently at 5 kGy or with 0.2% formalin (*v*/*v*) (Figure 1A,B). In addition, protein carbonylation, an irreversible oxidative protein modification, can disrupt the naïve protein structure, resulting in lower immunogenicity or an inappropriate immune response [39]. As shown in Figure 1C, the carbonyl-protein content of the prepared GBS lysate was determined, and the mean carbonyl values in all irradiated GBS samples were lower than 15 nmol/mg. In contrast, the levels of formaldehyde-inactivated GBS followed a strong concentration-dependent relationship. At a 0.2% concentration, the protein carbonyl levels reached 18 ± 2.1 nmol/mg, which was significantly higher than that obtained with 10 kGy-irradiated GBS. To compare the integrity of immunogenic epitopes between inactivated Rad-GBS and Che-GBS, the effective vaccine candidate peptide SRR1 and its reactivity in the prepared GBS was determined using an anti-SrrN2N3 monoclonal antibody via dot blotting [40]. GBS exposed to up to 15 kGy was still capable of binding strongly to the monoclonal antibody. Conversely, the formalin-inactivated samples lost most of their interactivity following exposure to 0.2% formaldehyde. The adsorption ability of the prepared GBS samples with rabbit anti-GBS serum was determined using ELISA (Figure 1D). Surprisingly, the reactivity of formalin-inactivated GBS (0.2%) was similar to that of irradiated GBS (10 kGy) (Figure 1E). The results obtained via ELISA also showed that Rad-GBS had a stronger adsorption affinity compared with Che-GBS, but was weaker compared with live GBS at 10^8^ and 10^9^ CFU/well (Supplementary Figure S1A–D). Taken together, these results suggested that Rad-GBS induced less damage to the surface epitopes, resulting in better immunogenicity compared to that with Che-GBS.

### 2.2. Rad-GBS Induces Maturation of Bone Marrow-Derived DCs 

**Figure 2:**
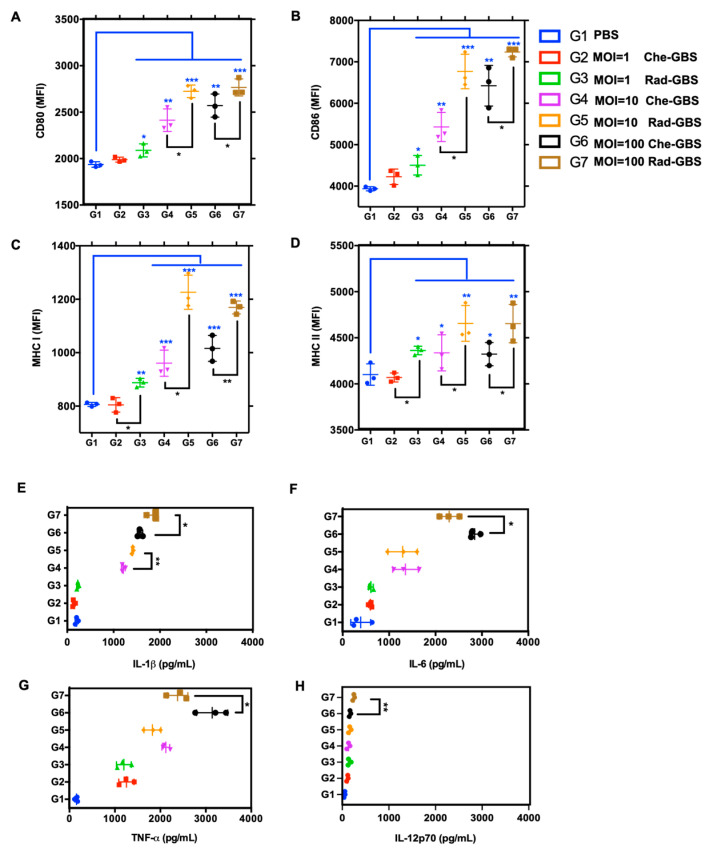
Rad-GBS enhances the activation of BM-DCs in comparison to that with Che-GBS. Bone marrow-dendritic cells (BM-DCs) (2 × 10^6^ cells/mL) were isolated and incubated with GM-CSF, followed by stimulation with either Rad-GBS or Che-GBS at 10^6^, 10^7^, and 108 CFU/well for 24 h. The expression of the co-stimulatory makers (**A**) CD80 and (**B**) CD86, (**C**) major histocompatibility complex MHCI, and (**D**) MHCII was analyzed via flow cytometry. The mean fluorescent intensity (MFI) is representative on the histograms. BM-DCs (2 × 106 cells/mL) were stimulated with Rad-GBS or Che-GBS at MOI for 24 h. Then, the levels of (**E**) IL-1β, (**F**) IL-6, (**G**) TNF-α, and (**H**) IL-12p70 in the culture supernatant were measured via ELISA. Scatter plots and other data suggest that MFI data are expressed as means ± SEM. All data are expressed as the mean ± standard deviation of triplicate samples or three independent experiments, and p-values were determined by performing an unpaired t test. *** Stands for *p* < 0.001, ** stands for *p* < 0.01, * stands for *p* < 0.05, and NS for not significant.

Dendritic cells (DCs) are essential for initiating adaptive immune responses through antigen presentation, which is dependent on the expression of co-stimulatory receptors and the major histocompatibility complex [41,42]. Following the stimulation of BM-DCs with either Rad-GBS or Che-GBS at various multiplicities of infection (MOIs) for 12 h, the mean fluorescence intensity (MFI) of several phenotypic maturation markers, including CD80 (*p* < 0.05), CD86 (*p* < 0.05), and MHCII (*p* < 0.05), was considerably increased upon stimulation with Rad-GBS (MOI = 1, 10, and 100), respectively (Figure 2A–D and Supplementary Figure S2). Notably, Rad-GBS at MOI = 100 induced the highest differential expression of MHCI (*p* < 0.05) compared to that with Che-GBS. This enhances pathogen peptide epitopes presented by MHCI for surveillance by the CD8^+^ T cell repertoire to induce cytotoxic immune responses [43]. Rad-GBS and Che-GBS potently induced pro-inflammatory cytokine secretion in the culture supernatants of BM-DCs stimulated with bacteria, which were collected for ELISA analysis. The results showed that Che-GBS and Rad-GBS dose-dependently increased the levels of IL-1β, IL-6, TNF-α, and IL-12p70. However, while Rad-GBS potently stimulated BM-DCs to produce IL-1β, it only marginally induced the production of IL-6, TNF-α, and IL-12p70 compared to that in those vaccinated with Che-GBS (Figure 2E–G). IL-1β is an essential mediator of the inflammatory response. It is involved in T cell proliferation and differentiation, and can provide mucosal adjuvant activity to induce sIgA immunity [44]. IL-12p70 plays an important role in DC maturation, such as efficient antigen uptake and presentation, and was significantly induced at an MOI of 100. These data suggested that Rad-GBS enhances the activation of adaptive cell-mediated responses by inducing BM-DC maturation, MHCI/II expression, and pro-inflammatory cytokines.

### 2.3. Rad-GBS Is Highly Immunogenic and Induces an Effective Humoral Immune Response

**Figure 3:**
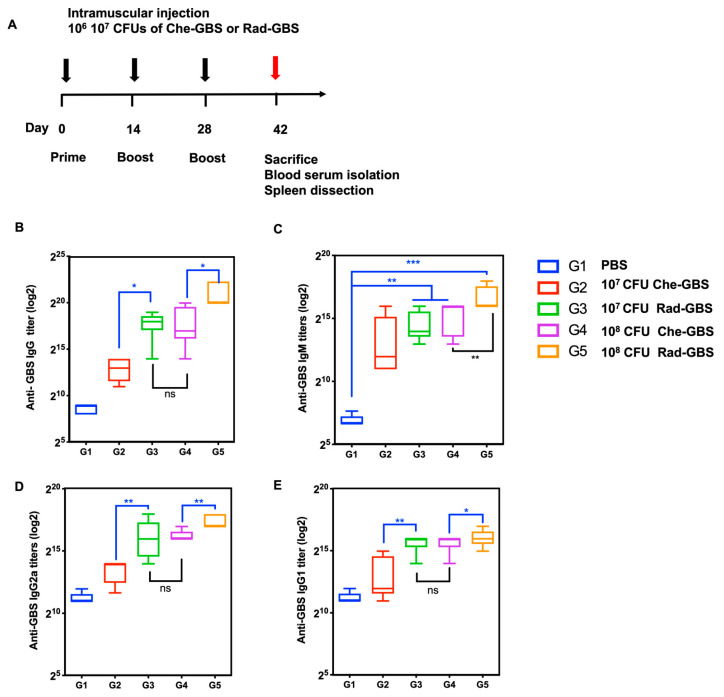
Immunoglobulin titers in sera from mice vaccinated with various dosages of Rad-GBS or Che-GBS. (**A**) Mice (*n* = 5) were vaccinated with 10^7^ or 10^8^ CFU of Rad-GBS or Che-GBS intramuscularly at two-week intervals, as in the schedule shown, and the mice were immunized with PBS as the control group. Serum levels of GBS-specific immunoglobulin G (IgG). (**B**) and immunoglobulin M (IgM) (**C**) were isolated and analyzed 7 days after the last immunization. The antibody levels of subclasses of IgG, such as (**D**) IgG1 and (**E**) IgG2a, were further determined. All data are expressed as the mean ± standard deviation of five mouse serum samples and three independent experiments. *** Stands for *p* < 0.001, ** stands for *p* < 0.01, * stands for *p* < 0.05, and NS for not significant.

To compare GBS-specific humoral immune responses between Rad-GBS and Che-GBS in vivo, mice (*n* = 5) were vaccinated with either 10^7^ or 10^8^ CFU of Rad-GBS or Che-GBS supplemented with 20 µg of aluminum gel adjuvant, as shown in Figure 3A. Primarily, humoral responses were determined by measuring immunoglobulin IgG and IgM titers in sera via ELISA. Rad-GBS at a high dose of 10^8^ CFU resulted in significantly increased levels of GBS-specific IgG (*p* < 0.05) and IgM (*p* < 0.01) titers compared to those with Che-GBS in mouse sera. However, at 10^7^ CFU, Rad-GBS elicited comparable IgG (288,000 ± 108,100) and IgM (25,600 ± 1238) antibody titers to those with 10^8^ of Che-GBS (Figure 3B,C). Rad-GBS elicited markedly higher IgG2a levels compared to those with Che-GBS in both the 10^7^ CFU (Rad-GBS: 76,800 ± 25,600, Che-GBS: 11,840 ± 5316) and 10^8^ CFU (Rad-GBS: 179,200 ± 62,706, Che-GBS: 86,400 ± 68,138) vaccinated groups, which may indicate a higher tendency towards a Th1-like immune response. Furthermore, the IgG1 subclass was more potently induced following administration of 10^7^ CFU of Rad-GBS and Che-GBS (Figure 3D,E). These data showed that Rad-GBS could potently induce IgG, particularly IgG2a, which provides protection against bacterial infection. 

### 2.4. Rad-GBS Induces T-Cell Dependent Immune Responses 

**Figure 4:**
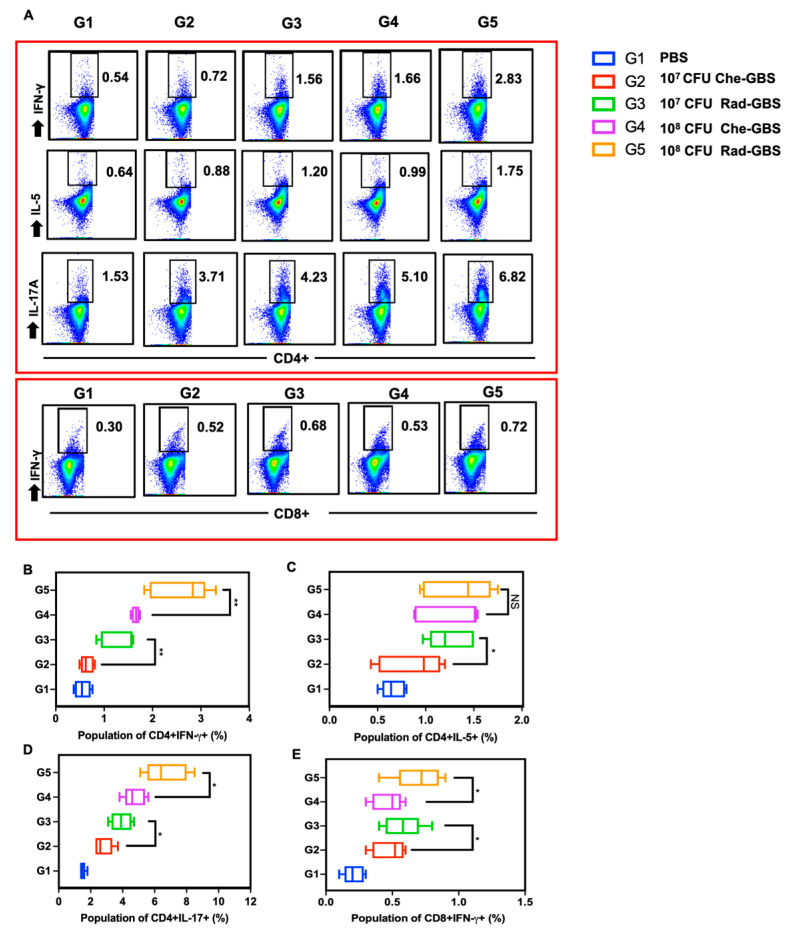
Cellular immunogenicity is potently elicited via immunization with Rad-GBS. Mice were intramuscularly immunized with various dosages of Rad-GBS or Che-GBS as shown above, and mice injected with PBS were used as the control group. Seven days after the last vaccination, the spleen cells were isolated, and cell suspensions were re-stimulated with 20 µg/mL GBS whole-cell lysate for 12 h and subsequently used for the flow cytometry analysis. (**A**) The functional populations of T cells were classified according to the expression of CD4^+^ and CD8^+^ of interferon gamma (IFN-γ), interleukin-5 (IL-5), or interleukin-17A(IL-17A) assessed in all mouse groups. Antigen-specific cytokine-producing population in isolated spleen cells. For mouse groups, measurements were made for the population of (**B**) CD4+IFN-γ^+^, (**C**) CD4^+^ IL5^+^, (**D**) CD4^+^ IL-17A^+^, and (**E**) CD8^+^ IFN-γ^+^. All data are expressed as the mean ± standard deviation of five splenocyte samples and three independent experiments. ** stands for *p* < 0.01, * stands for *p* < 0.05, and NS for not significant.

**Figure 5:**
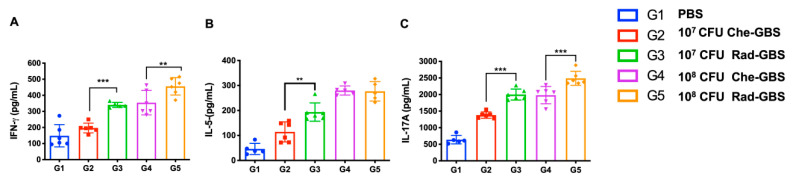
Cytokine production by spleen cells of vaccinated mice. Single-cell suspensions of spleen cells were isolated from mice above and were treated with 20 µg/mL GBS whole-cell lysate for 24 h. Supernatants were collected for the determination of cytokine levels, including (**A**) IFN-γ, (**B**) IL-5, and (**C**) IL-17A in the supernatant. All data are expressed as the mean ± standard deviation of five cell culture supernatant samples and three independent experiments, and p-values were determined by performing an unpaired t test. *** Stands for *p* < 0.001, ** stands for *p* < 0.01, and NS for not significant.

Activating specific T-cell immune responses is critical for cell-mediated immunity against bacterial pathogens [45]. To assess the activated cell-mediated immune responses in mice following immunization with Rad-GBS, cell suspensions of splenocytes were re-stimulated with GBS bacterial lysate, and the proliferation of IFN-γ^+^ CD4^+^, IL-5^+^ CD4^+^, and IL-17A^+^ CD4^+^ T cells was assessed (Figure 4A and Supplemental Figure S3). Both the Rad-GBS and Che-GBS groups had significant increases in T cells compared to the levels with PBS. Mice immunized with the relatively high doses Rad-GBS (10^8^ CFU) exhibited a significantly higher population of CD4^+^ IFN-γ^+^ (Rad-GBS: 2.58 ± 0.56, Che-GBS: 1.65 ± 0.08; *p* = 0.0045) and CD4^+^ IL-17A^+^ (Rad-GBS: 6.70 ± 1.32, Che-GBS: 4.74 ± 0.69; *p* = 0.019) T cells compared with those immunized with Che-GBS (Figure 4A–E). Mice immunized with 10^8^ CFU Rad-GBS showed no significant difference in CD4^+^ IL-5^+^ cells compared to levels in the Che-GBS group (Figure 4C). Interestingly, regardless of the doses (10^7^ or 10^8^ CFU), the population of CD8^+^ IFN-γ^+^ T cells in the Che-GBS group was barely activated (Figure 4D). This trend was also observed for cytokines in splenocyte culture supernatants; significantly higher levels of IFN-γ, IL-5, and IL-17A were detected in the Rad-GBS-immunized group compared with those in the PBS and Che-GBS-immunized groups. Notably, the levels of IL-17A in the Rad-GBS-immunized spleen cells were significantly higher than those in the control and Che-GBS groups (Figure 5C). Pathogen-specific IL-17 production was significantly stronger in mice immunized with Rad-GBS; a marked increase in IL-17 levels is likely to be relevant for protection against GBS mucosal infections. These data demonstrated that Rad-GBS induces a Th1/Th17 response in the current conventional formalin-inactivated vaccine.

### 2.5. Rad-GBS Confers Protection against Hypervirulent Strains in Mice

**Figure 6:**
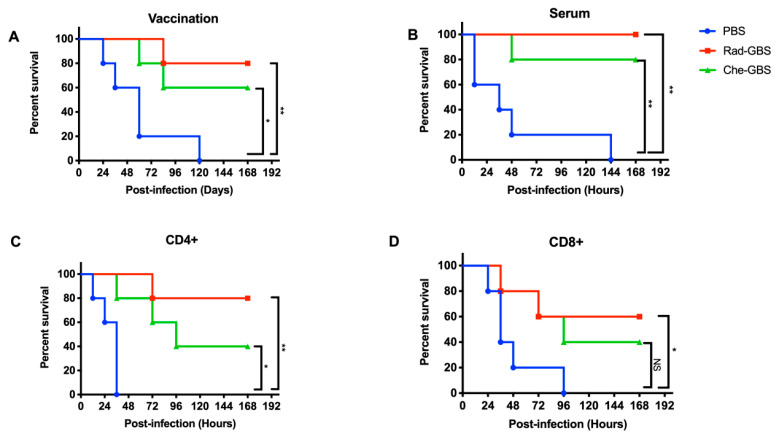
Evaluation of Rad-GBS-induced protection and increased survival in response to CNCTC 10/84 challenge. Mice (*n* = 5) were intramuscularly vaccinated with 10^8^ CFU of Rad-GBS or Che-GBS as described above. (**A**) All mice were intravenously challenged with 5 × 10^8^ CFU of CNCTC 10/84 GBS, and survival in each group was recorded daily for one week. To investigate the protection origins from immune responses, adoptive transfers of vaccinated mouse serum or CD4^+^or CD8^+^ T cells with GBS challenge was used. (**B**) Serum (300 μL), (**C**) Splenic CD4^+^ T cells, or (**D**) CD8^+^ T cells from alum or vaccinated mice was inoculated intraperitoneally into naïve C57BL/6 mice (*n* = 5). At 24 h following transfer, mice were also challenged intravenously with CNCTC 10/84 GBS. Mouse survival was monitored for 7 days. The survival of mice was determined using Kaplan–Meier survival analysis, and the significance of the difference was analyzed using a log-rank test. ** stands for *p* < 0.01 and * stands for *p* < 0.05 and NS for not significant.

To confirm that the GBS-specific immune responses elicited by Rad-GBS could provide strong protection, mice vaccinated with 10^8^ CFU of Rad-GBS or Che-GBS were challenged with 5 × 10^6^ CFU of hypervirulent GBS CNCTC 10/84 strains 7 days after the last immunization. The results indicated that immunization with Rad-GBS conferred 100% protection. In contrast, Che-GBS provided partial protection, with a survival rate of 80%. The survival rates of both vaccinated groups were significantly higher than that of the control group (Figure 6A). To investigate the mechanism through which Rad-GBS conferred protection, sera, CD4^+^, and CD8^+^ T cells were isolated from Rad-GBS- or Che-GBS-vaccinated mice and transferred to naïve mice. One day after transfer, all mice were challenged with the CNCTC 10/84 GBS strain. The results revealed that adoptively transferred CD4^+^ cells from Rad-GBS and Che-GBS mice markedly prolonged the lifespan of mice compared with that of the control group. However, the transfer of Rad-GBS sera conferred considerable protection against infection (Figure 6B–D). The survival rates of control mice or those transferred Che-GBS serum remained low, at 20 and 40% post-challenge, respectively, which were lower than that of the Rad-GBS group at 80%. Taken together, these data suggested that vaccination with Rad-GBS induced sufficient humoral and CD4^+^ T cell-dependent immune responses against GBS infection.

### 2.6. Vaccination with Rad-GBS Induces Functional Opsonic Killing Activity and Provides Cross-Protection against Heterologous Serotypes

**Figure 7:**
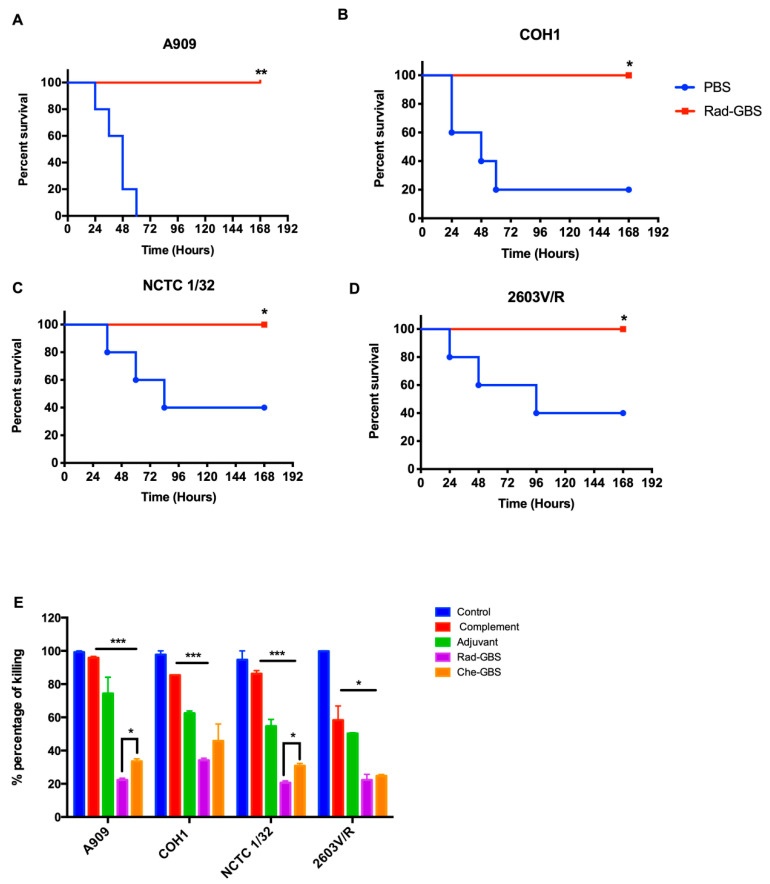
Cross-reactive protection highly induces functional antibodies against multiple serotypes via Rad-GBS vaccination. Mice (*n* = 5) were vaccinated intramuscularly with 10^7^ CFU of Rad-GBS supplemented with alum (20 µg), and the cohort was administrated PBS as a control. Mice were challenged intravenously with a lethal dose of GBS strains (**A**) A909 (Ia), (**B**) COH1 (III), (**C**) NCTC 1/32 (IV), and (**D**) 2603VR (V) at 7 days after the last immunization. Survival of the mice was observed and recorded for one week. (**E**) Opsonic killing activity of the sera isolated from Rad-GBS- or Che-GBS-immunized mice against multiple serotypes of GBS strains (A909, COH1, NCTC1/32, and 2603V/R) was examined. The differentiated HL-60 cells (4 × 10^6^ cells) and 10µL of baby rabbit complement were added into the mixed suspension of the diluted sera (1:500) isolated from the immunized mice and bacteria, while the negative control group contained only differentiated HL-60 cells and heat-inactivated baby rabbit complement. All data were expressed as the means ± standard deviation per group. The survival of mice was determined using Kaplan–Meier survival analysis, and the significance of the difference was analyzed using a log-rank test. *** Stands for *p* < 0.001, ** stands for *p* < 0.01, * stands for *p* < 0.05, and NS for not significant.

An ideal universal GBS vaccine should provide serotype-independent protection against multiple serogroups, in contrast to polysaccharide-conjugated vaccines. Rad-GBS-vaccinated mice received a lethal dose of the most prevalent GBS serotypes, including A909 (Ia), COH1(III), CNCTC 1/32, and 2603V/R(V). As shown in Figure 7A–D, Rad-GBS conferred 100% protection against each serotype, with no mortality recorded within 7 days. Control mice challenged with A909 (Ia) all died; other groups challenged with 2603V/R (V) or NCTC1/32 (IV) exhibited a 40% survival rate, while COH1 (III) exhibited a 20% survival rate at the end of the experiments. In addition, the opsonic activity of antisera from mice vaccinated with either Rad-GBS or Che-GBS conferred increased killing activity compared with that in the control and complement-only groups. However, compared to sera from the Che-GBS group, those from the Rad-GBS group had higher killing activity against A909 (23.35, 35.03%) and NCTC1/32 (21.80, 29.28%). Interestingly, the GBS 2603V/R strain was inhibited in the presence of antisera from the Rad-GBS and Che-GBS mice. However, non-specific potent opsonic killing was observed in the complement only group, with a survival rate 55.76% relative to that of the control group (Figure 7E). Taken together, these data indicated that Rad-GBS elicited functional antibodies that provided serotype-independent protection compared to Che-GBS, consistent with the previous upregulation of immunogenicity observed in vitro.

## 3. Discussion

GBS infections can result in newborn mortality and are common in the elderly and immunocompromised individuals [8,46]. Vaccine prophylaxis may provide an effective strategy for controlling GBS infections. Previous studies have shown that non-typeable GBS accounts for approximately 10% of all clinical isolates [47]. Despite the development of capsular polysaccharide-conjugate vaccines, which provide serotype-specific immunity, prevalent geographic serotype-switching and non-typeable clinical GBS threaten their use [16,48]. GBS vaccines should be developed for serotype-independent protection and cost-effectiveness. Inactivated vaccines are used widely for bacterial and viral prophylaxis and treatment and provide cross-reactive immunity [18,49]. This study investigated an irradiated GBS vaccine that provides homogeneous protection against most serogroups that activate early humoral responses.

Conventional chemical inactivation methods are limited by theoretical safety concerns, including the possibility of an increased risk of contamination and toxicity. Hazardous chemicals, such as formaldehyde or BPL, can induce conformational changes or irreversible damage to the antigenic epitopes of pathogens [19,50]. This modification decreases the humoral immune response by impairing the activity of influenza surface antigens hemagglutinin (HA) and neuraminidase (NA) [26]. In contrast, gamma radiation is considered superior to traditional inactivation approaches, because ionizing radiation can effectively penetrate pathogens and specifically eliminate nucleic acids while inducing minor damage to epitopes without increasing temperature and contamination chemicals [51]. Protein characterization of live and Rad-GBS using SDS-PAGE revealed no significant difference [52]. Nevertheless, as a vaccine candidate, we utilized a serine-rich repeat (SRR), which contains multiple immunologically relevant epitopes and is critical for facilitating GBS invasion by binding fibrinogen [32]. In the present study, Rad-GBS exhibited strong affinity for anti-SrrN2N3 monoclonal antibodies. The anti-GBS polyclonal antibody detected higher absorbance of the anti-GBS polyclonal antibody against GBS in Rad-GBS. The integrity and immunogenicity of SRR1 were inversely related to the concentration of protein carbonyl, thus indirectly explaining the reduced damage to the epitopes.

Previously, microwave-irradiated *Streptococcus agalactiae* was shown to elicit a GBS-specific humoral response and provided partial protection against Nile tilapia challenge [53]. Our results showed that the vaccination of mice with Rad-GBS induced greater protection than Che-GBS due to potent IgG antibody titers, particularly IgG2a and IgG2b, which correlate with CD4^+^ T cell activation. The humoral immunity and cell cytotoxicity elicited by Rad-GBS can be transferred to the naïve host to provide functional protective effects. Notably, the survival rate of sera-transferred mice was 100% post-challenge, indicating that mice sera confer good opsonic killing activity. However, CD4^+^ and CD8^+^ T cells isolated from Rad-GBS mice demonstrated only partial protection from lethal challenges and reduced the lifespan of the host mice. 

DC activation and antigen presentation following bacterial exposure are critical for eliciting adaptive immunity and initially activate T-cell dependent cytotoxic activity [54]. Previously, irradiated *S. pneumoniae* was found to upregulate DC expression of co-stimulatory molecules and MHC II in vitro, and the DCs effectively processed and presented exogenously administered antigens with BM-DCs compared to h-SP and f-SP [55]. In the present study, Rad-GBS significantly induced the maturation markers CD80 and MHCI in BM-DCs. Induction levels were higher than those of Che-GBS. The difference in the ability of Rad-GBS to induce the expression of maturation markers may be due to GBS persistently colonizing the genito-gastrointestinal tract rather than the pharyngeal tract, where DCs are loaded with other immune cells in lymphatic organs. Moreover, encapsulated GBS is highly internalized by DCs because the absence of capsules may also be related to surface antigen epitope recognition by pattern recognition receptors (PRR), leading to the upregulation of an adaptive immune response. In this study, Rad-GBS more potently induced IL-1β, IL-12p70, and IL-6 expression than did Che-GBS, which is believed to be correlated with Th1 and Th17 polarization in CD4^+^ T cells and has been widely used as a marker of vaccine responsiveness. These results suggest that Rad-GBS activates DCs, which efficiently present epitopes to T cells, leading to highly-induced adaptive immunity in vaccinated individuals. 

Cytotoxic T lymphocytes (CTL) are considered to be pivotal to the induction of immune memory and long-lasting protection against streptococcal infections [56]. Recent studies have demonstrated that radiation-inactivated *Salmonella galinarum* or *Streptococcus pneumoniae* confer strong protection via increased Th1- and Th17-mediated immunity [57]. The upregulation of CD4^+^ T cell profiles in response to GBS can be accessed via the production of a type 1 pro-inflammatory response, including the release of IFN-γ, TNF-α, and IL-5, and the recruitment of T cells via chemokines, such as CXCL9, CXCL10, and CCL3 [58]. In the present study, we revealed that both Rad-GBS and Che-GBS induced the proliferation of CD4^+^IFN-γ^+^; however, Rad-GBS preferentially enhanced the differentiation of CD4^+^ IL-17A^+^ T cells. This phenomenon has also been observed when irradiated *Streptococcus pneumoniae* and *Pseudomonas aeruginosa* were used for inactivated vaccines. [59,60]. IL-17 plays a protective role against intracellular and extracellular infections and a sentinel role in the rapid recruitment of neutrophils [61,62,63]. Another study found that IL-17A stimulated intestinal epithelial cells to enhance the production of antimicrobial proteins and chemokines. Thus, enhanced CD4^+^ Th1 and Th17-mediated immune responses might be responsible for the adequate protection elicited by Rad-GBS vaccination. 

Although capsular polysaccharide-based GBS vaccines remain dominant in terms of development and clinical trials, conjugate vaccines against *Streptococcus pneumoniae* and *Neisseria meningitidis* do not confer universal coverage for all prevalent serotypes. However, immune pressure and selection against these pathogens can be expected to change their natural habitat and serogroups in the post-vaccine period. Thus, the ecology of GBS may make the effects of polysaccharide-conjugate vaccines on epidemiology challenging to predict [64]. In this study, our findings suggested that Rad-GBS could activate sufficient adaptive immune responses, likely CD4^+^ T cell-dependent and humoral immunity. Our study also showed that gamma radiation-inactivated non-capsular Group B *Streptococcus* could be a promising vaccine candidate. Ultimately, this facilitates a strategic approach for the development of a universal candidate vaccine with effective serotype-independent protection against multiple capsule GBS serogroups. 

## 4. Materials and Methods

### 4.1. Bacteria and Culture 

All bacterial strains used in this study are listed in Table 1. Briefly, the non-capsulated *Streptococcus agalactiae* clinical isolate NSP14-358 was obtained at the Korea University Guro Hospital (Seoul, Republic of Korea). The other reference strains, A909, CNCTC10/84, COH1, 2603V/R, and NCTC 1/32, were kindly provided by Prof. Paul Sullam (University of California, San Francisco, CA, USA). All GBS strains were streaked on blood agar plates, grown overnight at 37 °C, and cultured in Todd Hewitt broth (THB; BD Biosciences, San Jose, CA, USA) supplemented with 0.2% yeast extract (BD Biosciences) at 37 °C. 

### 4.2. Preparation of Inactivated Bacteria 

Non-capsulated GBS NSP14-358 and its genome were sequenced and verified using the PacBio RS II platform (Pacific Biosciences, Menlo Park, CA, USA) at Macrogen Co., Ltd. The inactivated bacteria were prepared as previously described [58]. Bacteria were grown in Todd Hewitt broth (TH: Difco, BD Biosciences, Franklin Lakes, NJ, USA) at 37 °C until the mid-log phase was reached, as determined by the optical density (OD_600_ = 0.8–1.0). The cells were harvested via centrifugation at 4000 rpm for 20 min at 4 °C and then washed with phosphate-buffered saline (PBS pH = 7.2) twice before inactivation. Next, the cells were irradiated using a cobalt-60 gamma-ray irradiator (Advanced Research Technology Institute; Jeongeup, Republic of Korea) at an absorbed dose of 15 kGy for 1 h at 23 °C. Cells were then frozen in 30% glycerol and stored at −80 °C until use. For the preparation of formalin-inactivated GBS, equal numbers of cells were incubated with PBS containing 0.2% formaldehyde (*v*/*v*) (Sigma-Aldrich St, Merck KGaA, Louis, MO, USA) at 37 °C for 2 h with shaking. CHE-GBS was prepared via incubation with 0.2% formaldehyde solution (JUNSEI; Tokyo, Japan) under mild agitation at 23–28 °C for 2 h. To confirm the complete inactivation of GBS, cryovials were thawed and diluted bacteria were plated on blood agar plates (Ansan, Republic of Korea) and grown overnight at 37 °C under 5% CO_2_. 

### 4.3. Protein Carbonylation Assay

The bacteria were grown as described above. Aliquots of suspended cells were exposed to 1.5, 7.5, 10, and 15 kGy or incubated with PBS containing formaldehyde (0.1, 0.2, 1, 2, and 5%) for 2 h at 37 °C. Total bacterial protein samples were sonicated using a TECAN sonicator (TECAN, Osaka, Japan) for 5 min on ice. The samples were then centrifuged at 10,000× *g* for 15 min at 4 °C. Protein concentration and purity were determined by measuring the absorbance at 280 and 260 nm. Protein carbonylation was measured using a protein carbonyl colorimetric assay kit (Cayman, Ann Arbor, MI, USA) according to the manufacturer’s instructions. Briefly, 200 µL of the lysate was mixed with 800 µL 2,4-dinitrophenylhydrazine and 800 µL of 2.5 M HCl. The proteins were then precipitated using 20% trichloroacetic acid (TCA) at 4 °C for 5 min. After centrifugation, the pellet was washed with 1 mL of an ethanol and ethyl acetate mixture (1:1, *v*/*v*) and resuspended in 500 µL of guanidine hydrochloride. The optical density (OD) of the pellet was measured at 360–385 nm using a BIOTEX microplate reader.

### 4.4. Analysis of Epitope Integrity by Performing Dot Blots

Irradiated and formalin-inactivated GBS were analyzed for the preservation of antigenicity using a dot blot assay, performed as previously described [65]. The bacteria were grown as described above and inactivated with formalin. The bacterial suspension was adjusted to OD = 2.0. Diluted cell suspensions were spotted onto nitrocellulose membranes (Bio-Rad, Hercules, CA, USA) for drying. The membrane was then blocked by soaking it in 5% BSA in TBS-T (20 mM Tris-HCl, 150 mM NaCl, and 0.05% Tween-20, pH 7.5) and incubated with anti-rabbit Srr1N2N3-specific polyclonal antibody (1:1000; NEO Group. Inc, South Korea). Following incubation with primary antibodies, the membranes were incubated with anti-rabbit IgG conjugated with horseradish peroxidase-conjugated secondary antibody (1:5000; Sigma-Aldrich, St. Louis, MO, USA, Merck KGaA, Darmstadt, Germany) for 1 h at room temperature. Finally, the dot blots were developed using West Pico chemiluminescent substrate (Thermo Fisher Scientific, Waltham, MA, USA) and visualized with the Bio-Rad ChemiDoc™ Touch imaging system (Bio-Rad; Hercules, CA, USA) for data acquisition and analysis.

### 4.5. Analysis of Mouse Bone Marrow-Derived DC Phenotype

Bone marrow-derived dendritic cells (BM-DCs) were isolated and differentiated from 6–12-week-old C57BL/6 male mice (Orient Inc., Seongnam-si, Republic of Korea) as previously described [66]. Whole bone marrow cells from femurs and tibiae were lysed with red blood cell lysis buffer (Sigma-Aldrich, Merck KGaA). The bone marrow cells were differentiated into immature DCs using RMPI-1640 (GIBCO, Carlsbad, CA, USA) supplemented with 10% FBS (Biowest; Nuaille, France), 100 U/mL penicillin, 100 U/mL streptomycin, and GM-CSF (20 ng/mL; GreGene, South Korea) for 8 days. Culture media containing GM-CSF were added on days 4 and 7. The cells were harvested and analyzed on day 8. BM-DCs were stimulated with various concentrations of formalin-inactivated and radiation-inactivated GBS S9993 in RPMI-1640 containing GM-CSF (20 ng/mL; GreGene) for 16 h. The cells were then harvested and stained with conjugated monoclonal antibodies specific to CD80, CD86, and MHCI/II for 20 min on ice and washed with ice-cold PBS. The mean fluorescence intensity (MFI) and cell proportions were analyzed using a BD FACSverse flow cytometer (BD Bioscience) and FlowJo software (TreeStar Inc., San Carlos, CA, USA).

### 4.6. Mouse Experiment and Ethics 

Six-week-old C57BL/6 female mice were purchased from Orient Inc. (Suwon, Republic of Korea). Recommendations in the Guide approved all animal experiments for the Care and Use of Laboratory Animals of the National Institutes of Health. All animal experiments were approved by the Committee on the Use and Care of Animals at the Korea Atomic Energy Research Institute (KAERI; approval no. IACUC-2018-007) and performed according to the accepted veterinary standards set by the KAERI Animal Care Center. Mice were euthanized using a CO_2_ inhalation method, as specified by KAERI. 

Mice were randomly assigned to individually ventilated housing cages, with five mice per cage (OrientBio Inc., Seongnam-si, Republic of Korea). To evaluate immunological responses, all mice were immunized intramuscularly with 10^7^ or 10^8^ CFU of either Rad-GBS or Che-GBS, with aluminum hydroxide gel (20 µg; BRENNTG, Essen, Germany) as an adjuvant, in 100 μL of PBS three times at 2-week intervals. Seven days after the last immunization, all mice were euthanized, and the blood and spleen were isolated for further analysis. To investigate the protective efficacy of Rad-GBS and Che-GBS, mice were vaccinated as described above. Seven days after the last immunization, mice were inoculated intravenously with 5 × 10^6^ CFU of CNCTC 10/84 GBS, and mortality was observed and recorded daily for 1 week. To evaluate the cross-protection capacity of Rad-GBS, C57BL/6 mice (*n* = 5) were immunized intramuscularly with 10^7^ CFU of Rad-GBS supplemented with aluminum (20 µg; BRENNTG) three times at 2-week intervals. The group was vaccinated with alum only, as in the control group. Seven days after the last immunization, all control and vaccinated mice were administrated 5 × 10^6^ CFU of A909, 5 × 10^6^ COH1, or 1 × 10^7^ NCTC1/32 intravenously, and mortality was observed and recorded for 1 week.

### 4.7. Determination of Immunoglobulin in Sera

Blood samples were obtained from immunized C57BL/6 mice via cardiac puncture 7 days after the last immunization. Serum was isolated via centrifugation at 500× *g* for 10 min at 4 °C and stored at −80 °C for long-term use. The GBS was grown and harvested at mid-log phase and then sonicated for 15 min at 4 °C. Next, 100 μL lysate of the GBS pellet was adjusted to 0.1 at 600 nm by diluting it in PBS, used to coat 96-well immunoplates (SPL), and incubated overnight at 4 °C. The plates were washed five times with PBS-T, and the cells were then blocked with 1% BSA in PBS for 1 h at room temperature. After blocking, diluted sera were added to each well, after which the cells were incubated at room temperature for 1 h and then washed with PBS-T. The secondary antibodies were as follows: goat anti-mouse Ig-HRP (1:3000; Sigma-Aldrich, Merck KGaA), goat anti-mouse IgG-HRP (1:3000; SouthernBiotech, Birmingham, AL, USA), goat anti-mouse IgM-HRP (1:3000; SouthernBiotech), goat anti-mouse IgG1-HRP (1:3000; SouthernBiotech), goat anti-mouse IgG2a-HRP (1:3000; SouthernBiotech), goat anti-mouse IgG2b-HRP (1:3000; SouthernBiotech), and goat anti-mouse IgG3-HRP (1:3000; SouthernBiotech). The plates were then washed five times with PBS-T, after which 100 µL of 3,3′,5,5′-tetramethylbenzidine substrate reagent (BD Biosciences) was added. When the color developed, 50 µL of 2N H_2_SO_4_ was added, and the absorbance was measured at 450 nm using a Victor X3 light plate reader (PerkinElmer Inc., Waltham, MA, USA).

### 4.8. Opsonophagocytic Killing Assays (OPKAs)

Experiments were performed using HeLa-60 cells [67]. Briefly, the serum pool samples obtained from each mouse group were diluted (1:500) with buffer (OBB; Hanks’ buffer supplemented with 0.1% gelatin (Sigma-Aldrich, Merck KGaA) and 10% FBS in 96-well plates (SPL). Heat-inactivated complement (10 µL) was used as a negative control. Frozen GBS stocks (Table 1) were thawed immediately before use and washed twice with OBB, after which the cells were resuspended and adjusted to 10^5^ CFU/mL. Following incubation at room temperature for 30 min with shaking, 40 µL of a differentiated HL-60 cell suspension containing 4 × 10^5^ cells and 10 µL baby rabbit complement (BRC; Pel-Freez Biological: Rogers AR) was added. The cell mixture was incubated for 45 min at 37 °C with shaking. Next, 10 µL of the final mixture was spotted onto THY agar containing 1.5% Bacto agar (BD Biosciences). After drying, the plates were overlaid with 30 mL of THY agar containing 2% Bacto Gar. Surviving bacterial colonies on THY agar plates were counted. The percentage of opsonic activity = CFUs with complement and serum/CFUs control × 100%. All data were calculated from three independent experiments performed in triplicate. 

### 4.9. Flow Cytometry Analysis

Spleen homogenates obtained from vaccinated mice were filtrated through a 40 µm cell strainer (BD Biosciences) in RPMI-1640 medium (GIBCO, Carlsbad, CA, USA) supplemented with 10% fetal bovine serum (Biowest, Nuaille, France). All erythrocytes were lysed in RBS lysis buffer (Sigma-Aldrich, Merck KGaA) for 5 min and washed with RPMI-1640 medium. Cell suspensions, diluted to 2 × 10^6^ cells/mL, were seeded onto 48-well cell culture plates (SPL) and stimulated with 20 µg/mL of GBS NSP 14-358 lysate in the presence of 0.5 µg/mL GolgiStop (eBioscience, San Diego, CA, USA) and 0.5 µg/mL GlogiPlug (eBioscience) for 12 h at 37 °C. The cells were washed with cold PBS and stained for T cell-surface markers using a LIVE/DEAD staining kit (Aqua vitality dye, InvivoGen, San Diego, CA, USA), anti-CD4-BV421 (1; 200), and anti-CD8a (1; 200) antibodies (BD Bioscience) and then washed in cold PBS. The cell suspensions were subsequently permeabilized using a Cytofix/Cytoperm kit (BD Bioscience) for 30 min at 4 °C. Finally, the cells were stained intracellularly with anti-IFN-γ (PE, BD Biosciences), anti-IL-5 (APC, BD Biosciences), and anti-IL17A (PE-Cy7, BD Biosciences) antibodies. All stained cells were analyzed using a BD FACSverse flow cytometer (BD Biosciences).

### 4.10. Cytokine Measurements

To analyze extracellular cytokine production, suspensions of splenocytes from each immunized mouse were isolated and stimulated with 20 µg/mL GBS NSP 14-358 lysate for 72 h at 37 °C. IFN-γ, TNF-α, and interleukin-5 levels were measured using an enzyme-linked immunoassay kit (BD Bioscience). All procedures were performed in accordance with the manufacturer’s instructions.

### 4.11. Adoptive Transfer of T Cells and Serum into Naïve Mice 

To isolate CD4^+^ and CD8^+^ T cells, mouse spleen cells were isolated and perfused with RPMI-1640 medium. The procedures were performed according to the manufacturer’s instructions (Miltenyl Biotec, Bergisch Gladbach, Germany). Briefly, the splenocytes were centrifuged at 300× *g* for 5 min and suspended in 40 μL buffer (PBS pH 7.2, supplemented with 1 mM EDTA) containing 10^7^ cells. Then, 10 μL of a biotin–antibody cocktail was added, and the cells were incubated for 10 min at 4 °C. Next, 30 μL of buffer and 20 μL of anti-biotin microbeads were added per 10^7^ cells. The cells were further incubated for 15 min at 4 °C. The cells were then washed with 1–2 mL buffer and centrifuged at 300× *g* for 5 min, after which the supernatant was discarded, and the cell pellets were resuspended in buffer. The total cell mixture was transferred to an MS column (Miltenyl Biotec) to purify the CD4^+^ and CD8^+^ cells. Six-week-old C57BL/6 female mice were randomly distributed into six experimental groups of five mice each and then used for passive transfer experiments. Serum or T cells (CD4^+^ or CD8^+^) from immunized mice were transferred to naïve mice via intraperitoneal (*i.p.)* injection. The serum volume and number of transferred cells were approximately 300 µL and 1 × 10^6^ T cells, respectively, per mouse. At 24 h post-passive transfer, all mice were infected with 5 × 10^7^ CFU of the CNCTC 10/84 hypervirulent strain. The survival rates of mice in each group were monitored for 14 days after infection.

### 4.12. Statistical Analysis

All results are expressed as the mean ± standard deviation of more than triplicate samples or three independent experiments. All data in the bar and dot graphs between groups were compared using an unpaired Student’s t-test for normal data distribution or the Mann–Whitney test for non-parametric variables (the values under consideration are random, and are independent of each other, as are the observations within each sample). The survival of mice was determined using Kaplan–Meier survival analysis, and the significance of the difference was analyzed using a log-rank test. The results with p-values less than 0.05 were considered statistically significant; *** stands for *p* < 0.001, ** stands for *p* < 0.01, * stands for *p* < 0.05, and NS for not significant. Graphs and statistical analyses were performed using ver. 6.0 (GraphPad Software, Inc., La Jolla, CA, USA).

## Figures and Tables

**Table 1:** Group B streptococcus strains used in this study.

Name	Serotype	Characteristics	Reference or Source
A909	Ia	Wild-type	[31]
COH1	III	Wild-type	[31]
NEM316 ΔSrr1	III	Wild-type, serine-rich repeat 1 protein knockout	[32]
NCTC 1/82	IV	Wild-type	[33]
CNCTC 10/84	V	Wild-type, hypervirulent	[34]
2603 V/R	V	Wild-type	[35]
NSP14-358	Non-typeable	Clinical isolate from urine of a 74-year-old female with levofloxacin-resistant infection	[36]

## Data Availability

Data is contained within the article and supplementary material.

## Supplementary Materials

The following supporting information can be downloaded at: https://www.mdpi.com/article/10.3390/ph16020321/s1, Figure S1: Rad-GBS showed higher adsorption in comparison with Che-GBS; Figure S2: Rad-GBS modestly induces activation of BM-DCs in comparison with Che-GBS; Figure S3: Flow cytometric analysis of functional CD4+ and CD8+ T cells in mice immunized with Rad-GBS or Che-GBS.
