# Supplementary material for: Gamma-Irradiated Non-Capsule Group B Streptococcus Promotes T-Cell Dependent Immunity and Provides a Cross-Protective Reaction
Source: Pharmaceuticals (Basel). 2023 Feb 20;16(2):321. doi: 10.3390/ph16020321 (PMC9961543; doi:10.3390/ph16020321)
Supplement: Supplementary file 1 [file pharmaceuticals-16-00321-s001.zip › pharmaceuticals-2204240-supplementary.pdf]

Gamma-irradiated Non-Capsule Group B Streptococcus Promotes the T-cell dependent Immunity and confers a Cross protection

Yong Zhi[1], Fengjia Chen[2], Guangxu Cao[1], Fang Li[1]

1. Department of Obstetrics and Gynecology, Shanghai East Hospital, School of Medicine, Tongji University, Shanghai, 200092, China

2. Research Division for Radiation Science, Korea Atomic Energy Research Institute, Jeongeup 56212, Jeollabuk-do, Republic of Korea

Supplementary Material

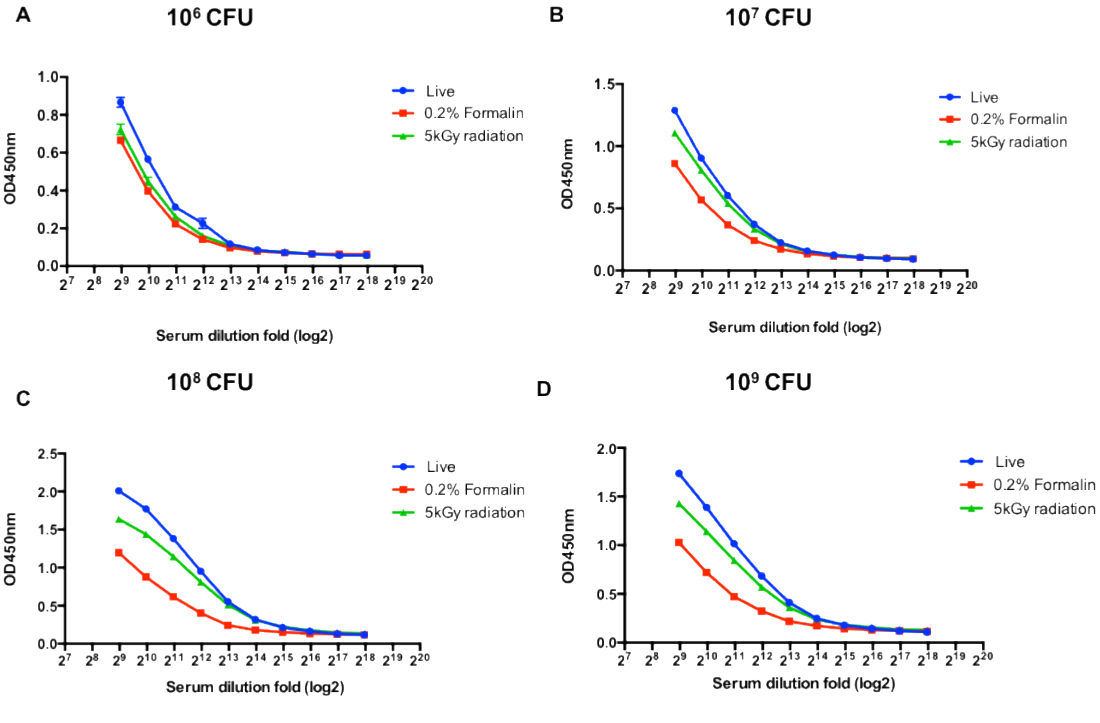

Supplementary Figure 1. Rad-GBS showed higher adsorption in comparison with Che-GBS. The constant numbers of inactivated GBS (A)10^6 CFU/well (B)10^7 CFU/well, (C) 10^8CFU/well or (D) 10^9 CFU/well were coated on immunoplate, anti- GBS serum were directly added and serially diluted, after incubation for 45 min at room temperature. The optical density absorbance at (OD450)

are measured and representative of three independent experiments and are presented as the mean ± standard deviation.

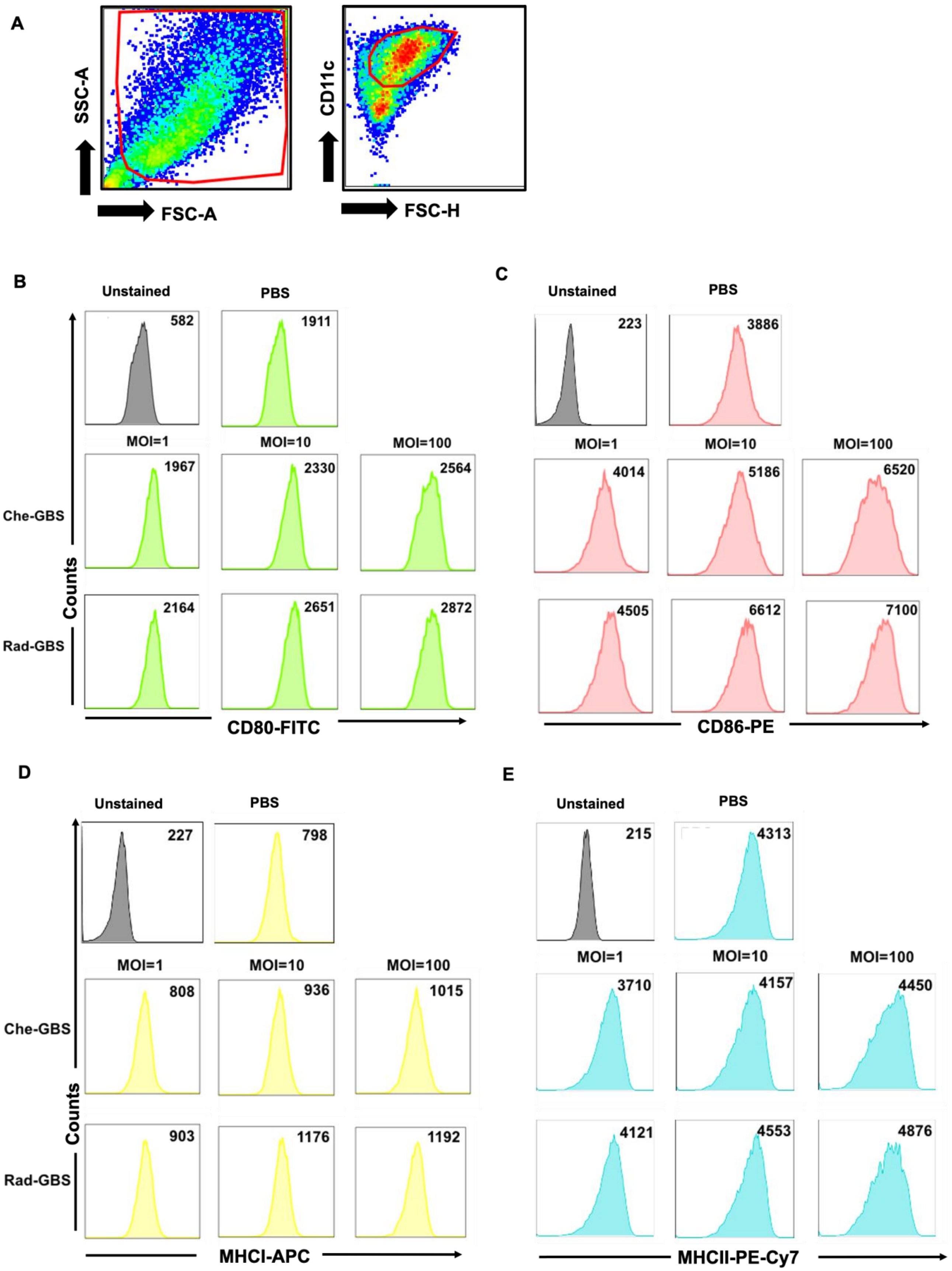
A
SSC-A
FSC-A
CD11c
FSC-H
B
Unstained
PBS
582
1911
MOI=1
MOI=10
MOI=100
1967
2330
2564
Che-GBS
Counts
2164
2651
2872
Rad-GBS
CD80-FITC
C
Unstained
PBS
223
3886
MOI=1
MOI=10
MOI=100
4014
5186
6520
4505
6612
7100
CD86-PE
D
Unstained
PBS
227
798
MOI=1
MOI=10
MOI=100
808
936
1015
Che-GBS
Counts
903
1176
1192
Rad-GBS
MHCI-APC
E
Unstained
PBS
215
4313
MOI=1
MOI=10
MOI=100
3710
4157
4450
4121
4553
4876
MHCII-PE-Cy7

Supplementary Figure 2. Rad-GBS modestly induces activation of BM-DCs in comparison with Che-GBS. Bone marrow-dendritic cells (BM-DCs) (2×10^6 cells/mL) were isolated and incubated with GM-CSF, followed by stimulation with either Rad-GBS or Che-GBS at 10^6, 10^7, and 10^8 CFU/well for 24h. The expression of the co-stimulatory markers CD80 and CD86, major histocompatibility complex MHCI and MHCII was analyzed by flow cytometry.

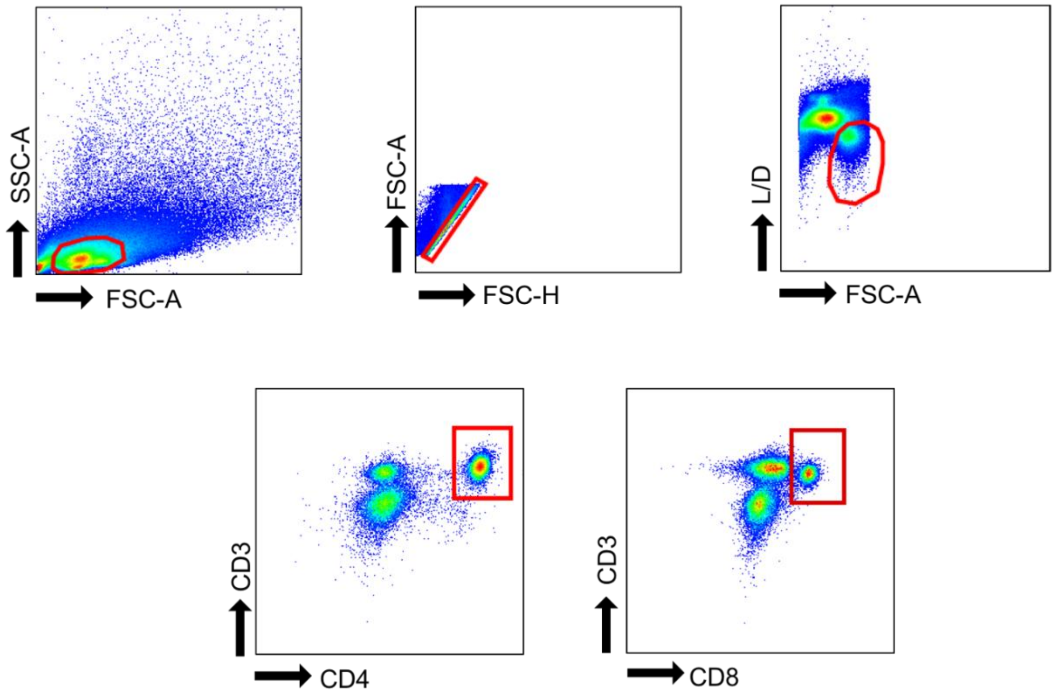

Supplementary Figure 3. Flow cytometric analysis of functional CD4+ and CD8+ T cells in mice immunized with Rad-GBS or Che-GBS. Mice (n=5) were immunized with various dosages of Rad-GBS or Che-GBS as scheduled, and mice injected with alum were only used as the control

group. Functional T cells were classified and gated according to the expression of CD4+ or CD8+ on the cell surface.
